# Patient factors influencing acute gluten reactions and cytokine release in treated coeliac disease

**DOI:** 10.1186/s12916-020-01828-y

**Published:** 2020-11-26

**Authors:** Jason A. Tye-Din, A. James M. Daveson, Kaela E. Goldstein, Holly L. Hand, Kristin M. Neff, Gautam Goel, Leslie J. Williams, Kenneth E. Truitt, Robert P. Anderson, on behalf of the RESET CeD Study Group, A. Adams, J. Andrews, C. Behrend, G. Brown, S. Chen Yi Mei, A. Coates, A. J. Daveson, A. DiMarino, H. Ee, D. Elliott, R. Epstein, B. Feyen, R. Fogel, K. Friedenberg, R. Gearry, M. Gerdis, M. Goldstein, V. Gupta, R. Holmes, G. Holtmann, S. Idarraga, G. James, T. King, T. Klein, S. Kupfer, B. Lebwohl, J. Lowe, J. Murray, E. Newton, D. Quinn, D. Radin, T. Ritter, H. Stacey, C. Strout, R. Stubbs, S. Thackwray, V. Trivedi, J. A. Tye-Din, J. Weber, S. Wilson

**Affiliations:** 1 Immunology Division, The Walter and Eliza Hall Institute, Parkville, VIC Australia; 2 Department of Medical Biology, University of Melbourne, Parkville, VIC Australia; 3 Department of Gastroenterology, The Royal Melbourne Hospital, Parkville, VIC Australia; 4 Coral Sea Clinical Research Institute, Suite 7, 76 Willetts Road, North Mackay, QLD 4740 Australia; 5 University of Queensland, Brisbane, QLD Australia; 6 ImmusanT Inc., Cambridge, MA USA; 7 Wesley Medical Research, PO Box 499, Toowong, QLD 4066 Australia

**Keywords:** Coeliac disease, Gluten, Food challenge, Cytokine, Patient-reported outcome

## Abstract

**Background:**

Patients with coeliac disease (CD) commonly report a variety of adverse symptoms to gluten, but descriptions of the symptomatic response in the literature may have been confounded by the presence of food components such as fermentable carbohydrates (FODMAPs) causing symptoms of irritable bowel syndrome independent of gluten. In recent unmasked and masked low FODMAP gluten challenge studies in small groups of treated CD patients, nausea and vomiting were shown to be the key symptoms associated with serum interleukin (IL)-2 release. Our objective was to utilise a large and diverse cohort of people with CD undertaking a standardised gluten food challenge to characterise the demographic, genetic and clinical factors influencing the severity and timing of acute gluten reactions and IL-2 production.

**Methods:**

A total of 295 adults treated for CD were observed for 6 h after an unmasked food challenge consisting of 10 g vital wheat gluten (low in FODMAPs) in 100 ml water. Assessments included patient-reported outcomes, serum IL-2 and adverse events. Responses were analysed according to patient characteristics, HLA-DQ genotype, duodenal histology and response to a second gluten challenge.

**Results:**

Peak symptom severity was at 3 h (median severity 5/10). Peak IL-2 was at 4 h (median 4 pg/ml, range undetectable to 1028 pg/ml). Older age, older age at diagnosis, HLA-DQ2.5 positivity and homozygosity for HLA-DQB1*02 were each significantly associated with IL-2 elevations after gluten. Patients positive for HLA-DQ2.5, DQ8, DQ2.2 or DQ7 showed elevated IL-2 after gluten. Patient factors were not significantly associated with severity of digestive symptoms, but symptoms were correlated to one another and serum IL-2. Gluten challenge after 5 months caused more vomiting and higher IL-2 levels, but responses correlated with the first.

**Conclusions:**

Gluten-induced symptoms and cytokine release is common in adults with treated CD. Age, genetics and previous response to gluten predict these acute reactions to gluten challenge. Structured symptom assessment and serum IL-2 after standardised gluten challenge may inform on patient diagnosis, the role of gluten in symptomatology and the need for adjunctive treatment.

**Trial registration:**

ClinicalTrials.gov, NCT03644069 Registered 21 May 2018.

## Background

Coeliac disease (CD) is a prevalent, small intestinal immune-mediated enteropathy precipitated by exposure to dietary gluten in genetically predisposed people [1, 2]. Patients with CD commonly report adverse symptoms associated with gluten ingestion [3], but there are few studies that rigorously control for confounding effects to enable accurate gluten-related symptomatology to be recorded. These include nocebo effects, where patient’s negative expectations to potential gluten exposure influence their symptoms and the presence of non-gluten dietary components such as fermentable carbohydrates (FODMAPs) that can cause symptoms of irritable bowel syndrome (IBS) independent of gluten [4, 5]. In several recent gluten food challenge studies in small groups of patients with treated CD, unmasked and also double-blind, sham-controlled gluten challenges designed to be low in FODMAPs cause significant worsening of nausea, sometimes with vomiting, within 2-h and peak at 3 to 4 h, but rarely caused diarrhoea [6, 7]. This acute symptomatic reaction to gluten in patients with treated CD is linked to significant concomitant elevations in serum cytokines, which are not observed in individuals without CD or those with self-reported non-CD gluten sensitivity [7–9].

Serum levels of interleukin (IL)-2 increase earliest and most in the serum cytokine signature observed after gluten [10]. An almost identical set of digestive symptoms and systemic cytokine release occurs after patients with treated CD receive an intradermal injection of gluten peptides corresponding to immuno-dominant HLA-DQ2.5-restricted epitopes for gluten-specific CD4+ T cells [8, 11]. Collectively, these findings implicate an unexpected leading role for activated gluten-specific CD4+ T cells in the early symptoms after gluten. However, more detailed understanding requires studies in substantially larger cohorts that are not focused on patients positive for HLA-DQ2.5 alone.

The objective of the present study was to characterise demographic, genetic and clinical factors influencing the severity of symptoms and IL-2 release after gluten in a large cohort of patients having a standardised, low FODMAP gluten challenge. The data provide a roadmap outlining the potential clinical role for bolus gluten challenge in the care of patients with and without CD following a gluten-free diet.

## Methods

### Study design

**Fig. 1:**
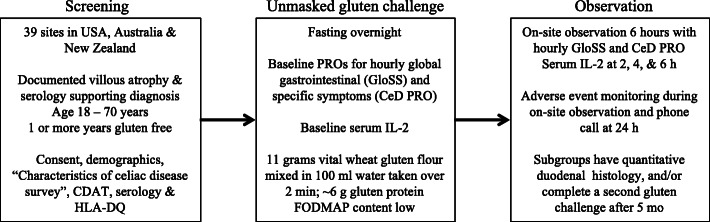
Study outline

The study design is shown in Fig. 1. Here, we report data collected from 295 CD individuals during the screening (pre-treatment) phase of the “RESET CeD Study”, a phase 2 clinical trial of an investigational treatment for CD (ClinicalTrials.gov identifier: NCT03644069). Full details of the RESET CeD Study design are described elsewhere [6]. There were 39 sites in the USA, Australia and New Zealand. All participants undertook an unmasked (i.e. open-label) gluten food challenge coupled with an assessment of IL-2 and symptom responses. A later component of the RESET CeD Study following the administration of the experimental drug/placebo involved CD participants completing a double-blind food challenge of gluten at the same dose and format as undertaken during screening or a matched non-gluten placebo, each low in FODMAPs and consumed 2 weeks apart. The data from the 36 CD patients who received placebo drug helps control for nocebo effects associated with open-label administration of gluten and is reported elsewhere [6].

### Participants and study procedures

All patients gave written, informed consent prior to undergoing any trial-related procedures. Full eligibility criteria are listed elsewhere [6]. To enter screening, patients’ ages were between 18 and 70 years, they had documentary evidence of duodenal villous atrophy and elevated CD-specific serology and had followed a gluten-free diet for at least 1 year. Patients were excluded from the analysis if their HLA-DQ genotype was subsequently found to be incompatible with CD (absence of HLA-DQA1*05, HLA-DQA1*03, HLA-DQB1*02 or HLA-DQB1*03), or no symptom data had been recorded after screening gluten challenge. Unmasked gluten challenge was as described by Tye-Din et al. [7]. The composition was as described by Daveson et al. [6]. The food challenge, taken as a bolus in 100 ml water over 2 min, comprised 10 g vital wheat gluten (Gem of the West® Vital Wheat Gluten, Manildra Group, Auburn, NSW, Australia). The protein content of the vital wheat gluten was 78 g per 100 g (Eurofins Microbiology Laboratories Inc). FODMAP content was low, with a total fructan of 0.074 g/serve and a total FODMAP of 0.089 g/serve using reported methods [12] (Monash University, Melbourne, Victoria, Australia).

### Clinical assessments

Demographics, medical history and prior and concomitant medications were recorded. Patients completed the characteristics of CD survey described by Tye-Din et al. [7] and the Celiac Dietary Adherence Test (CDAT) [13]. As described elsewhere [7], self-reported symptom severity was recorded within an hour before, and then each hour up to 6 h after gluten challenge using a modified version of the “Celiac Disease Patient-Reported Outcome” (CeD PRO®) tool, and using a global gastrointestinal symptom survey (GloSS). Adverse events during the 24 h after gluten challenge were separately recorded and graded by investigators according to Common Terminology Criteria for Adverse Events, version 4.03.

### Laboratory assessments

CD serology (QUANTA Lite® “R h-tTG IgA” and “gliadin IgG II (DGP)”, INOVA Diagnostics, San Diego, CA, USA) was performed by ARUP (Salt Lake City, UT). HLA-DQ genotype was evaluated by the UCLA Immunogenetics Center (Los Angeles, CA). The blood for IL-2 was allowed to clot for 30 min and, within 3 h, aliquots of the serum were frozen and stored at − 60 to − 80 °C. Serum IL-2 was measured by a commercial V-PLEX electrochemiluminescence assay as previously described [6]. The lower level of quantitation was 0.5 pg/ml.

### Statistical analyses

We utilised non-parametric tests as we did not assume a normal distribution for the data. The Mann-Whitney test was used to compare two datasets of continuous variables that were unpaired, and for paired data, the Wilcoxon signed rank test and Spearman’s coefficient of correlation were used. Kruskal-Wallis testing was used to compare groups of three or more continuous variables in the same participant. Associations between categorical variables were tested by the Fisher exact test, chi-square test or Cochran-Armitage test depending on whether two variables, more than two variables or an order-dependent trend in variables was present, respectively. All statistical tests were 2-tailed, and a *p* value of 0.05 was taken as significant. Benjamini-Hochberg’s false discovery rate method was used to adjust *p* values for multiple comparisons where appropriate. Summary and significance statistics were computed using Graphpad Prism V7.0d. The overall clinical severity of a reaction to gluten for an individual from the least (“no symptoms”) to the worst (“very severe symptoms”) was deemed to be their worst recorded hourly GloSS descriptor over the 6-h interval after gluten.

## Results

### Patient characteristics

In total, data for 295 patients who had gluten challenges were analysed, which represented 77% of the 383 volunteers who were screened, and 95% of all the patients who were eligible and had a screening gluten challenge over the period from August 2018 to March 2019. Disposition of patients is shown in Additional file 1: Figure S1. The gluten challenge was on the first day of screening for 214 patients, or between 1 and 31 days after starting the screening for 96 patients. Demographics and characteristics of patients are shown in Table 1.

**Table 1:** Patient characteristics

Mean (SD) age in years	43 (15)
Number (%) of females	205 (69)
Mean (SD) height in centimetres	169 (11)
Mean (SD) body mass in kilogrammes	79 (19)
Mean (SD) body mass index	28 (13)
Number (%) of White, not Hispanic or Latino	277 (94)
Median (interquartile range) age in years at diagnosis of CD	35 (25–46)
Median (interquartile range) years from coeliac diagnosis	6 (3–10)
Number (%) of negative for both CD-specific serologies^†^	241 (82)
Number (%) of positive for both CD-specific serologies^†^	5 (2)
Number (%) of IgA deficient (< 7 mg/dl)	2 (1)
Number (%) of positive for HLA-DQ2.5 genotype	266 (90)
Number (%) of negative for HLA-DQ2.5 genotype	29 (10)
Number (%) of recruited in the USA	126 (43)
Number (%) of recruited in Australia	123 (42)
Number (%) of recruited in New Zealand	46 (16)

^†^QUANTA Lite® R h-tTG IgA (normal range 3 U/ml or less) and gliadin IgG II (DGP), INOVA Diagnostics (normal range 19 U or less)

### Patient-reported outcomes after gluten challenge

**Fig. 2:**
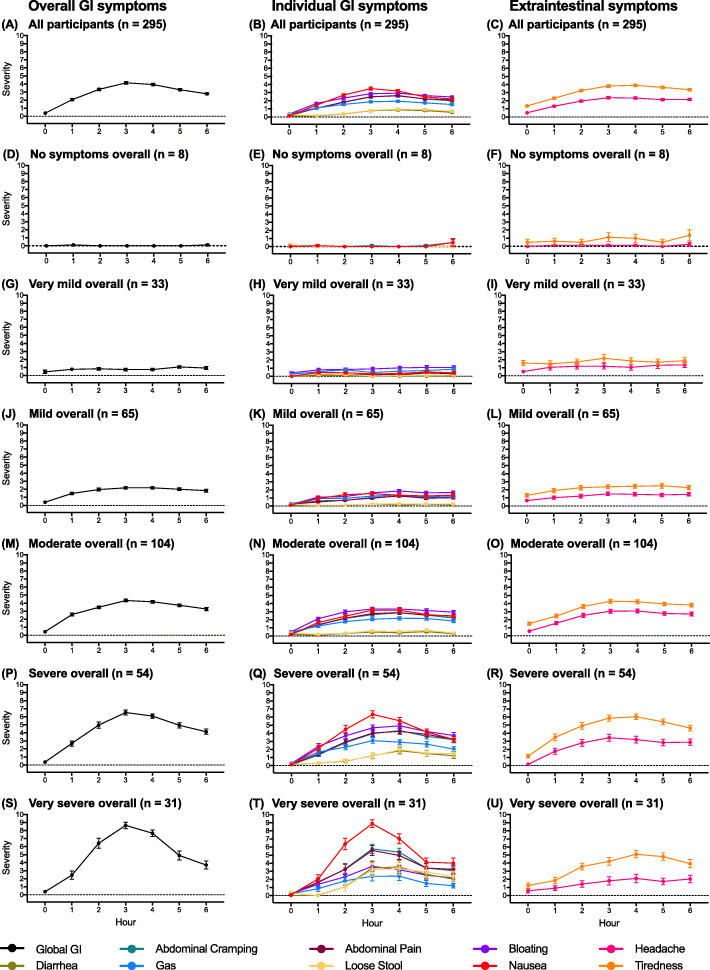
Six-hour time series after gluten for patient-reported outcome scores (modified CeD PRO and Global Symptom Survey). Profiles are for all patients (**a**–**c**) and for patients whose worst global gastrointestinal score over 6 h was “no symptoms” (**d**–**f**), “mild symptoms” (**g**–**i**), “very mild symptoms” (**j**–**l**), “moderate symptoms” (**m**–**o**) and “severe symptoms” (**p**–**r**), “very severe symptoms” (**s**–**u**). Points indicate mean ± standard error of the mean for scores rated 0 to 10

There was a good agreement between verbal descriptor and numerical score for peak hourly GloSS rating (Spearman *r* = 0.92, *p* < 10^−15^, *n* = 295) (Additional file 1: Table S1). Table 2 shows the distribution of peak GloSS severity and CeD PRO severity for individual symptoms. The overall severity of reactions based on peak GloSS descriptor was moderate and rated 5 on a scale from 0 to 10. Gluten reactions were graded moderate, severe or very severe by almost two thirds of patients. Figure 2 shows the time course for GloSS and CeD PRO ratings for global and individual symptoms, respectively. We again observed that patients’ expectations of symptoms were often not those experienced after gluten challenge (Additional file 1: Tables S2, S3) [6]. Onset was within 1 h, and peak overall severity of moderate, severe and very severe reactions was clearly at 3 h. Nausea, cramping and abdominal pain, bloating and tiredness steadily worsened as the overall severity of reactions increased, but diarrhoea and loose stool were only prominent in very severe reactions. Additional file 1: Table S4 shows that the peak severity of all individual symptoms assessed by the CeD PRO was correlated with one another. The strongest correlations were between the most prominent symptoms such as nausea, pain, cramping, bloating and tiredness. Table 3 shows how often patients rated individual symptoms among their three worst during the 6 h after gluten challenge. Nausea, tiredness and abdominal pain/cramping were each rated among the worst symptoms for almost half of the patients, but nausea and vomiting were clearly most often the worst symptoms for patients having very severe reactions.

**Table 2:** Peak severity of symptoms up to 6 h after consuming gluten

	Gluten reaction severity^‡^						All
	None	Very mild	Mild	Moderate	Severe	Very severe	
Number of patients (%)	8 (3)	33 (11)	65 (22)	104 (35)	54 (18)	31 (10)	295
**Median peak hourly severity of digestive symptoms rated each hour in the Global Symptom Survey**^**†**^ **(interquartile range)**							
Absolute value	0 (0–0)	2 (1–2)	2 (1–2)	6 (5–6)	8 (8–8)	10 (9–10)	5 (3–8)
Change from baseline	0 (0–0)	1 (1–2)	1 (1–2)	5 (5–6)	8 (7–8)	10 (9–10)	5 (3–7)
**Median peak hourly symptom score in modified CeD PRO**^**†**^ **(interquartile range)**							
Nausea	0 (0–0)	1 (0–2)	2 (1–4)	5 (3–6)	8 (7–9)	10 (10–10)	4 (2–8)
Diarrhoea	0 (0–0)	0 (0–0)	0 (0–0)	0 (0–1)	1 (0–6)	8 (1–10)	0 (0–3)
Loose stool	0 (0–0)	0 (0–0)	0 (0–1)	0 (0–3)	2 (0–8)	8 (2–10)	0 (0–3)
Cramping	0 (0–1)	1 (0–1)	2 (1–3)	4 (2–5)	7 (4–8)	8 (6–9)	4 (1–6)
Pain	0 (0–1)	1 (0–1)	2 (1–3)	4 (3–5)	6 (5–8)	8 (6–9)	4 (1–6)
Tiredness	2 (0–3)	2 (1–3)	3 (2–5)	6 (4–7)	7 (7–8)	6 (5–8)	5 (3–7)
Bloating	0 (0–0)	2 (1–2)	3 (2–3)	5 (3–6)	7 (5–7)	5 (3–7)	4 (2–6)
Gas	0 (0–0)	1 (1–2)	2 (1–3)	3 (2–4)	4 (2–6)	4 (1–7)	2 (1–4)
Headache	0 (0–0)	1 (0–3)	2 (0–4)	4 (2–6)	3 (1–8)	2 (1–6)	3 (1–5)

^‡^Peak overall severity was the worst hourly descriptor rating reported by patients over 6 h after gluten challenge in the Global Symptom Survey for the question, “Overall, how would you rate the severity of your digestive symptoms in the past 1 h? (e.g. feeling or being sick, abdominal pain or cramps, feeling bloated, having loose stools or passing gas)”

^†^Whole number rating scale from 0 for no symptoms to 10 for worst possible symptoms

**Table 3:** Number (%) of participants reporting a symptom as among their three most troubling symptoms after gluten challenge according to peak reaction severity

	Peak severity of global gastrointestinal symptoms (GloSS)^**‡**^						Total^**b**^
	None^**a**^	Very mild^**a**^	Mild^**a**^	Moderate^**a**^	Severe^**a**^	Very severe^**a**^	
Total patients	8 (3)	33 (11)	65 (22)	104 (35)	54 (18)	31 (11)	295 (100)
Nausea	1 (13)	5 (15)	29 (45)	54 (52)	32 (60)	21 (72)	142 (49)
Tiredness	3 (38)	16 (48)	32 (50)	50 (49)	24 (45)	3 (10)	128 (44)
Abdominal pain/cramps	1 (13)	8 (24)	24 (38)	48 (47)	24 (45)	13 (45)	118 (41)
Bloating	0	15 (45)	22 (52)	43 (42)	15 (28)	1 (3)	107 (37)
Headache	1 (13)	14 (42)	24 (38)	44 (43)	16 (30)	4 (14)	103 (36)
Vomiting	0	0	1 (2)	13 (13)	24 (45)	26 (90)	64 (22)
Gas	0	8 (24)	25 (39)	21 (20)	6 (11)	0	60 (21)
Diarrhoea/loose stool	0	1 (3)	5 (8)	12 (12)	11 (21)	14 (48)	43 (15)
None	5 (63)	1 (3)	0	1 (1)	0	0	7 (2)
Others	0	2 (6)	3 (5)	1 (1)	0	0	6 (2)

Participants’ responses to Q3 in the Global Symptom Survey, 6 h after gluten challenge, “What symptom/s overall have troubled you the most today, since having the food challenge?” Please select a maximum of 3 symptoms: A. none, B. abdominal pain/cramps, C. vomiting, D. nausea, E. diarrhoea/loose stool, F. gas (flatulence), G. bloating, H. tiredness, I. headache, J. others (please specify)

^‡^The peak overall severity was the worst hourly descriptor rating reported by patients over 6 h after gluten challenge in the Global Symptom Survey for the question, “Overall, how would you rate the severity of your digestive symptoms in the past 1 h? (e.g. feeling or being sick, abdominal pain or cramps, feeling bloated, having loose stools or passing gas)”

^a^Percentage of patients for each severity subgroup, e.g. none and very mild

^b^Percentage of all 295 patients

### Adverse events after gluten challenge

The timing, prevalence, and severity of adverse events were generally consistent with patient-reported outcomes (Additional file 1: Table S5). Vomiting affected 62 (21%) patients and was the only common adverse event not among those included in the CeD PRO. There were no serious adverse events, but 37 (13%) patients experienced a severe adverse event. Nausea and vomiting accounted for the majority of adverse events graded severe. The timing of onset for adverse events indicated a consistent march of symptoms beginning with nausea.

### Serum IL-2 after gluten challenge

**Fig. 3:**
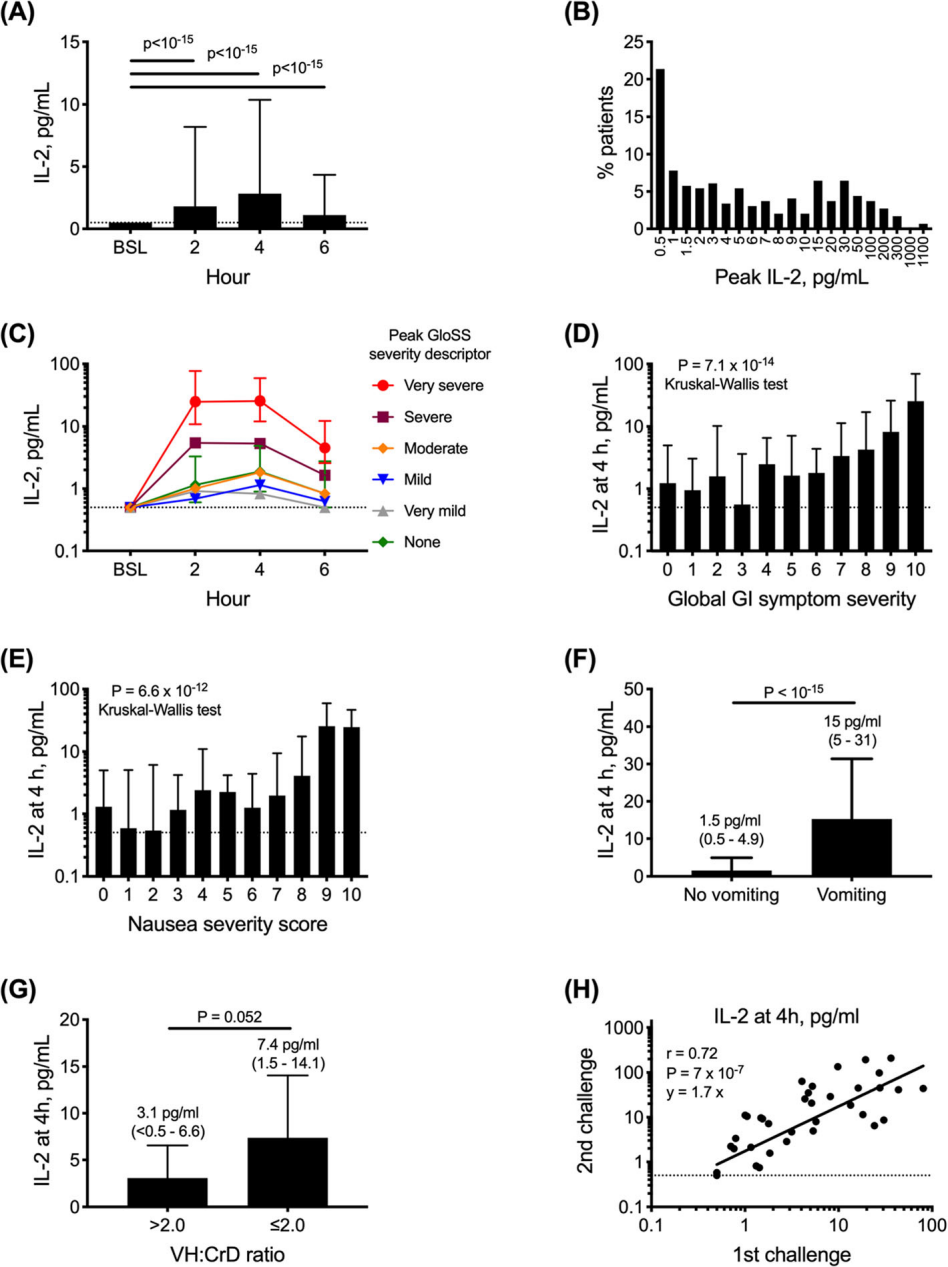
Serum IL-2 elevation in 295 patients over 6 h after gluten challenge. **a** Median and interquartile range of serum IL-2 concentrations at 2-h intervals after gluten; significance tested by Wilcoxon matched-pairs signed rank test. **b** The frequency distribution of peak serum IL-2 concentrations in patients after gluten. **c** Median and interquartile range of serum IL-2 concentrations at 4 h and peak hourly numerical rating for global severity of digestive symptoms (GloSS). **d** Median and interquartile range of serum IL-2 concentrations 4 h and peak hourly severity of nausea. **e** Median and interquartile range of serum IL-2 concentrations at 4 h and whether patients experienced vomiting after gluten challenge. **f** Mean and standard error of the mean of serum IL-2 concentrations after gluten according to gluten reaction severity (peak severity descriptor of digestive symptoms rated each hour in the GloSS). **g** Peak serum IL-2 concentrations after gluten according to villous height to crypt depth ratio (VH:CrD) in the second part duodenal biopsies indicating the presence of villous atrophy (VH:CrD ≤ 2) (*n* = 39 patients) or absence (*n* = 13). **h** Paired serum IL-2 concentrations at 4 h after two separate food challenges with 6-g gluten protein. Data for 36 patients who received placebo treatment during the RESET CeD Study and had both a screening (unmasked) and a second (masked) challenge 20 to 22 weeks later. The gluten challenges used the same format administering the equivalent of 6-g gluten protein in 10-g vital wheat gluten mixed in water

Serum IL-2 significantly increased by 2 h and typically peaked at 4 h (Fig. 3a). Altogether, serum IL-2 levels were elevated at 4 h in 216 (73%) patients who had baseline levels below 0.5 pg/ml. In addition, there were isolated elevations of IL-2 at 2 h in seven patients and at 6 h in four patients. Therefore, the total number of patients who elevated serum IL-2 from baseline to levels above the lower level of quantitation or at least 1.9 times more than the baseline was 229 (78%).

The median peak serum IL-2 concentration was 4 pg/ml (interquartile range 0.7–13), and the median peak fold change from baseline was 7 (interquartile range 1.2–25). The peak serum IL-2 levels were not normally distributed (Fig. 3b). The highest peak serum IL-2 concentration observed was 1075 pg/ml. Two (1%) patients had peak IL-2 levels above 1000 pg/ml, 15 (5%) had peak IL-2 levels above 100 pg/ml and 73 (25%) had peak IL-2 levels above 10 pg/ml, whereas serum IL-2 remained below 0.5 pg/ml in 63 (21%) patients. Serum IL-2 levels were above the lower level of quantitation (0.5 pg/ml) at baseline in five (2%) patients.

### Correlation between symptoms and IL-2 after gluten challenge

The serum IL-2 concentration at 4 h was correlated most strongly with peak GloSS score (Additional file 1: Table S4, Fig. 3c), and serum IL-2 time profiles were elevated most and for longer in very severe reactions (Fig. 3d). Nausea was the symptom most correlated with peak serum IL-2 levels (Fig. 3e), but correlations were also found with the severity of abdominal pain, abdominal cramping, bloating, loose stool and diarrhoea (Additional file 1: Table S4). Patients who vomited after gluten had median serum IL-2 levels at 4 h that were ten times higher than other patients (Fig. 3f). Among the 65 patients who did not show any elevation in serum IL-2, their peak GloSS severity was rated very mild in 11 (17%), mild in 21 (31%), moderate in 27 (42%) and severe in 4 (6%). Comparing patients who had no detectable elevation of IL-2 with patients who did, only nausea (median 3 vs 5, *p* = 0.004), global symptoms (median 4 vs 6, *p* = 0.0009) and vomiting (1 vs 60, *p* = 0.000024) were different. Interestingly, all eight patients who reported no symptoms had elevations in serum IL-2 levels from below 0.5 pg/ml at baseline to between 0.8 and 6.9 pg/ml.

### Associations between patient characteristics and gluten reactions

Additional file 1: Table S6 lists patient characteristics and their associations with peak global gastrointestinal symptom severity and with peak serum IL-2 after gluten challenge. None of the ten patient characteristics assessed was significantly associated with clinical severity. In contrast, patient age and age at diagnosis were associated with elevation in serum IL-2. In the subgroup of 52 patients described elsewhere who had quantitative histology performed on second part duodenal biopsies [14], patients with villous atrophy (Fig. 3g), or increased intra-epithelial lymphocyte (IEL) density, tended to have higher serum IL-2 levels at 4 h after gluten (IELs > 25 per 100 epithelial cells, *n* = 42, median 5.3 pg/ml, interquartile range 1.4–12.8 vs IELs ≤ 25, *n* = 10: 0.9, < 0.5–6.5; *p* = 0.083 by Mann-Whitney *U* test).

HLA-DQ genotype was associated with peak serum IL-2 concentration and the proportion of IL-2 responders, but not clinical severity of gluten reactions (Table 4, Additional file 1: Table S6 and S7). The median peak serum IL-2 concentration was more than seven times higher in patients positive for HLA-DQ2.5 than those who were negative (Table 4). Peak serum IL-2 concentrations were also significantly higher in homozygotes for HLA-DQB1*02 than heterozygotes. In the group of patients negative for HLA-DQ2.5, there were individuals positive for HLA-DQ8, HLA-DQ2.2 or HLA-DQ7 alone who elevated serum IL-2 levels and were symptomatic after gluten challenge (Additional file 1: Table S8). Most patients heterozygous for HLA-DQ8 who did not carry HLA-DQ2.2 or DQ7 did not elevate serum IL-2 after gluten. A very severe clinical reaction and strong serum IL-2 response was observed in one patient who was positive for HLA-DQ7 and HLA-DQ6.

**Table 4:** HLA-DQ genotype and serum IL-2 after gluten

Genotype group^†^, participants (*n*)	Serum IL-2 concentrations, median (interquartile range) pg/ml				Responders^‡^ participants, *n* (%)
	2 h	4 h	6 h	Maximum^††^	
1. DQ2.5 positive (266)	2.2 (< 0.5–8.6)	3.2 (0.6–10.7)	1.2 (< 0.5–4.5)	4.6 (0.8–13.1)	217 (82%)
2. DQ2.5 negative^‡‡^ (29)	< 0.5 (< 0.5–1.2)	0.6 (< 0.5–2.9)	< 0.5 (< 0.5–1.1)	0.6 (< 0.5–3.6)	17 (52%)
3. DQ2.5, 2.5 (34)	2.7 (0.8–8.0)	3.7 (1.3–9.4)	1.6 (0.6–4.9)	5.9 (1.8–10.8)	29 (85%)
4. DQ2.5, 2.2 (56)	5.5 (2.5–12.9)	9.0 (2.6–22.9)	3.7 (0.9–7.0)	10.2 (4.7–24.5)	52 (93%)
5. DQ2.5, 7 (15)	0.8 (< 0.5–7.4)	1.8 (< 0.5–5.0)	0.8 (< 0.5–1.4)	1.8 (< 0.5–9.0)	11 (73%)
6. DQ2.5, 8 (36)	1.1 (< 0.5–6.7)	2.4 (< 0.5–5.7)	0.9 (< 0.5–2.8)	2.7 (0.6–8.5)	28 (78%)
7. DQ2.5trans (18)	1.5 (0.7–8.2)	3.3 (0.9–12.5)	1.5 (0.6–6.9)	3.3 (0.9–12.5)	16 (89%)
8. DQ2.5cis, X (107)	1.4 (< 0.5–6.3)	1.9 (< 0.5–8.1)	0.8 (< 0.5–3.0)	2.5 (0.6–10.4)	81 (76%)

^†^*HLA-DQA* and *DQB* alleles for the following genotype groups: 1. DQA1*05 and DQB1*02 both present; 2. absent either or both DQA1*05 and DQB1*02; 3; homozygous both DQA1*05 and DQB1*02; 4. DQA1*02,05, and DQB1*02 homozygous; 5. DQA1*05 homozygous and DQB1*02,03; 6. DQA1*03,05 and DQB1*02,03; 7. DQA1*02,05 and DQB1*02,03; 8. Other patients with DQA1*05 and DQB1*02

^‡^Responders defined by maximum serum IL-2 concentration at least 0.5 pg/ml

^††^Mann-Whitney test false discovery rate-adjusted *p* values for six comparisons were significant for the presence of DQ2.5 (group 1 vs 2), *p* = 0.004; DQB1*02 gene dose (groups 5, 6, 7 and 8 vs 3 and 4), *p* = 0.0009; *p* values were not significant for DQA1*05 gene dose (groups 4, 6, 7 and 8 vs 3 and 5), DQ2.5 homozygotes vs DQ2.5, 2.2 (group 3 vs 4), DQ2.5cis, X vs DQ2.5trans (group 7 vs 8) and for DQ2.5,8 vs DQ2.5cis, X (group 7 vs 8)

^‡‡^See Table S8 for genotypes, symptoms and IL-2 responses of HLA-DQ2.5-negative patients

### Second gluten challenge after 5 months

A group of 36 placebo-treated patients had a masked gluten during the treatment period of the RESET CeD Study [6], which was between 20 and 22 weeks after their screening gluten challenge. Although the compositions of the two gluten challenges were identical, the second gluten challenge more often caused vomiting (16, 44% vs 8, 22%; *p* = 0.047, Fisher exact test), and serum IL-2 levels at 4 h were higher (median 10.1, 3.0–43 pg/ml vs 4.6, 1.3–17.8; *p* = 0.0013 Wilcoxon signed rank test). Individual patients’ serum IL-2 levels at 4 h after the first and second challenges were, however, closely correlated (Fig. 3h), and all but one patient who vomited after the first gluten challenge also vomited after the second (odds ratio [OR] 14.8; *p* = 0.012, Fisher exact test). A more detailed symptom analysis was not possible because patient-reported outcomes were assessed only after 1 day following the second gluten challenge [6].

## Discussion

This is the largest study to characterise symptoms and cytokine release following acute gluten exposure in CD. Due to its large sample and participant diversity, this study provides novel insights into the relation between gluten-mediated symptoms and immune activation, and the influence of patient factors. For the first time, we provide functional evidence for the influence of HLA-DQB*02 gene dose on in vivo immune activation after oral gluten ingestion. We also provide functional data supporting population studies that HLA-DQ7 should be considered among genotypes conveying susceptibility to CD [15, 16].

The present report replicates and greatly extends findings from our earlier studies involving smaller, more selected patient cohorts [6–8]. Here, we show that the severity of individual digestive symptoms was strongly correlated with one another and with elevations in serum IL-2. The kinetics of serum IL-2 levels matched the changing severity of early digestive symptoms, especially nausea. Some symptoms in the CeD PRO including gas, headache and tiredness were not correlated with serum IL-2 levels and, therefore, may be more strongly influenced by non-gluten factors, such as a nocebo effect.

In this study, cytokine release was reproducible after successive gluten challenges, and responses were closely correlated for individual patients. Therefore, it is likely that in patients with treated CD who inadvertently ingest gluten, the small intestinal mucosa, the gut-associated lymphoid organs and extra-intestinal organs are regularly exposed to elevated concentrations of pro-inflammatory cytokines. Systemic cytokine release after gluten may offer an explanation for some of the extra-intestinal manifestations reported for CD.

Fever, hypotension and hypoxia are cardinal features of the cytokine release syndromes associated with systemically administered agents [17], but these adverse events were absent or rare after gluten. This study shows that a single one-off gluten exposure transiently elevates serum IL-2 levels to as high as 1000 pg/ml, which is higher than the peak levels after the first dose of an anti-CD3 biologic [18] or with CAR T cell immunotherapy [19].

This study had several limitations. Although the number of patients enrolled in this study was more than eight times larger and more diverse than in previous bolus gluten challenge studies [6–8], our findings may not be relevant to children and adolescents, patients regularly consuming gluten or those without CD who strictly avoid gluten. In particular, participants in this study had volunteered for an experimental immunotherapy trial and may not be representative of patients with CD in the communities where they lived. A potentially significant limitation of this study was also an important strength: the food challenge had a standardised format with a fixed amount of gluten from a single supplier and a confirmed low FODMAP content. Findings may not be the same for different amounts of gluten administered in other formats. Our previous studies, however, indicate that masked and unmasked food challenges with the same amount and format as the present study result in comparable elevations in serum IL-2 [6, 7]. The gluten challenge dose is likely to be much higher than the amount patients with CD following gluten-free diet might be exposed to with accidental or inadvertent exposure. For logistical reasons, we measured a single cytokine in serum to quantify immune activation after gluten. Other cytokines may be useful to complement IL-2, but data available at present do not clearly indicate which other cytokines could serve this role [10]. The present study included all 36 patients reported separately following placebo food challenge in the treatment period of the RESET CeD Study [6].

The relative size and responsiveness of the gluten-reactive CD4+ T cell population, and the level of antigenic stimulation it receives, are an attractive explanation for the severity of symptoms and peak levels of serum IL-2 after gluten challenge [8]. Previously, we reported significant correlations between peak IL-2 levels and the frequency of tetramer-stained gluten-specific effector memory CD4+ T cells in the blood [8]. Factors that were significantly associated with higher serum IL-2 elevation after gluten in the current study included higher IL-2 response after a previous gluten challenge and HLA-DQ genotype being positive for HLA-DQ2.5 and homozygous for HLA-DQB1*02. The data presented here is the first in vivo evidence supporting a HLA-DQ2.5 gene dose effect on the intensity of immune reactivation after patients ingest gluten. CD participants who were HLA-DQ8 (without HLA-DQB1*02) had much lower IL-2 responses after gluten, consistent with the weaker association between this genotype and CD [20, 21]. Future assessment of this uncommon CD subgroup is warranted. Here, we also observed that older patients and older patient age at diagnosis were associated with greater IL-2 elevations. Future studies including children as well as older patients could address this intriguing observation.

The current study showed that gluten challenge with assessments of symptom severity and serum IL-2 may inform the care of patients following a gluten-free diet. For example, in those on a gluten-free diet with an unclear diagnosis of CD, a positive IL-2 result would strongly support a CD diagnosis whilst a negative IL-2 could identify those who need a diagnostic re-evaluation of CD. In confirmed CD, the degree of symptoms and IL-2 rise following a standardised challenge may help stratify the patients who would benefit most from adjunctive pharmaceutical treatments currently under development. Prospective validation to define the clinical utility of this approach is warranted.

## Conclusions

Most patients with treated CD experience a characteristic cytokine and symptom response after acute oral gluten challenge. Nausea and vomiting are hallmarks for moderate or more severe cytokine release reactions after gluten. Bolus gluten challenge with IL-2 assessments has the potential to be integrated into clinical practice to evaluate the diagnosis and management of patients on gluten-free diets.

## Supplementary Information

**Additional file 1: Table S1.** Number (%) of participants using a qualitative descriptor and corresponding numerical score for peak global digestive symptom severity (GloSS) after gluten challenge. **Table S2.** Patients’ ability to predict their most troubling symptoms after gluten. **Table S3.** Indication for testing, worst symptoms at diagnosis, and worst symptom expected after gluten exposure on gluten free diet, number (%). **Table S4.** Correlations between peak severity of symptoms and IL-2 at 4 hours after gluten. **Table S5.** Adverse events within 24 h after gluten challenge. **Table S6.** Symptom severity and IL-2 after gluten according to patients’ characteristics. **Table S7.** Peak symptom severity after gluten according to HLA-DQ genotype. **Table S8.** Symptoms and serum IL-2 after gluten in 29 participants negative for HLA-DQ2.5 (DQA1*05 and DQB1*02). **Table S9.** Study Independent Ethics Committees and approvals. **Figure S1.** Patient disposition.

## Data Availability

The datasets used and/or analysed during the current study are available from the corresponding author on reasonable request.

## Abbreviations

CDAT: Celiac Dietary Adherence Test
CeD PRO®: Celiac Disease Patient-Reported Outcome
FODMAP: Fermentable oligo-, di-, monosaccharides and polyols
GloSS: Global Gastrointestinal Symptom Survey
HLA: Human leukocyte antigen
