# Supplementary material for: Patient factors influencing acute gluten reactions and cytokine release in treated coeliac disease
Source: BMC Med. 2020 Nov 26;18:362. doi: 10.1186/s12916-020-01828-y (PMC7690153; doi:10.1186/s12916-020-01828-y)
Supplement: Supplementary file 1 — Additional file 1: Table S1. Number (%) of participants using a qualitative descriptor and corresponding numerical score for peak global digestive symptom severity (GloSS) after gluten challenge. Table S2. Patients’ ability to predict their most troubling symptoms after gluten. Table S3. Indication for testing, worst symptoms at diagnosis, and worst symptom expected after gluten exposure on gluten free diet, number (%). Table S4. Correlations between peak severity of symptoms and IL-2 at 4 hours after gluten. Table S5. Adverse events within 24 h after gluten challenge. Table S6. Symptom severity and IL-2 after gluten according to patients’ characteristics. Table S7. Peak symptom severity after gluten according to HLA-DQ genotype. Table S8. Symptoms and serum IL-2 after gluten in 29 participants negative for HLA-DQ2.5 (DQA1*05 and DQB1*02). Table S9. Study Independent Ethics Committees and approvals. Figure S1. Patient disposition. [file 12916_2020_1828_MOESM1_ESM.docx]

**Supplementary materials – Additional File 1**

| **Table S1:** Number (%) of participants using a qualitative descriptor and corresponding numerical score for peak global digestive symptom severity (GloSS) after gluten challenge**†** | | | | | | | | | |
| --- | --- | --- | --- | --- | --- | --- | --- | --- | --- |
| Score |  | Qualitative descriptor | | | | | |  | Total |
|  |  | None | Very mild | Mild | Moderate | Severe | Very severe |  |  |
| 0 |  | 7 (88) | 0 | 0 | 0 | 0 | 0 |  | 7 (2) |
| 1 |  | 1 (13) | 15 (45) | 1 (2) | 0 | 0 | 0 |  | 17 (6) |
| 2 |  | 0 | 11 (33) | 7 (11) | 0 | 0 | 0 |  | 18 (6) |
| 3 |  | 0 | 4 (12) | 30 (46) | 3 (3) | 0 | 0 |  | 37 (13) |
| 4 |  | 0 | 2 (6) | 16 (25) | 11 (11) | 0 | 0 |  | 29 (10) |
| 5 |  | 0 | 1 (3) | 7 (11) | 35 (34) | 0 | 0 |  | 43 (15) |
| 6 |  | 0 | 0 | 3 (5) | 40 (38) | 4 (7) | 0 |  | 47 (16) |
| 7 |  | 0 | 0 | 0 | 12 (12) | 11 (20) | 0 |  | 23 (8) |
| 8 |  | 0 | 0 | 0 | 3 (3) | 26 (48) | 1 (3) |  | 30 (10) |
| 9 |  | 0 | 0 | 1 (2) | 0 | 7 (13) | 10 (32) |  | 18 (6) |
| 10 |  | 0 | 0 | 0 | 0 | 6 (11) | 20 (65) |  | 26 (9) |
| Total |  | 8 (3) | 33 (11) | 65 (22) | 104 (35) | 54 (18) | 31 (11) |  | 295 (100) |
| Median  (IQR) |  | 0  (0 - 0) | 2  (1 - 2) | 3  (3 - 4) | 6  (5 - 6) | 8  (7 - 8.25) | 10  (9 - 10) |  | 5  (3 - 7.5) |
| † Spearman correlation coefficient comparing nominal and descriptor for peak GloSS severity, r = 0.92, p < 10^-15^ (n = 295) | | | | | | | | | |

| **Table S2:** Patients’ ability to predict their most troubling symptoms after gluten† | | | | | |
| --- | --- | --- | --- | --- | --- |
| Symptom expected to be worst after gluten | Number of patients  n (%) | Predicted correctly  n (%) | Predicted  incorrectly  n (%) | Odds ratio†† | P value†† |
| Any symptom**‡** | 282 (96) | 145 (49) | 137 (47) | 1.06 | Not applicable |
| Pain | 83 (28) | 45 (54) | 37 (45) | 1.22 | 1 |
| Diarrhoea | 66 (22) | 11 (17) | 53 (80) | 0.13 | 1.8 x 10^-9^ |
| Vomiting | 39 (13) | 26 (67) | 13 (33) | 2.08 | 0.17 |
| Bloating | 31 (11) | 25 (81) | 6 (19) | 4.55 | 0.0031 |
| Fatigue | 18 (6) | 13 (72) | 4 (22) | 3.27 | 0.18 |
| Headache | 15 (5) | 8 (53) | 7 (47) | 1.08 | 1 |
| Nausea | 13 (4) | 12 (92) | 1 (8) | 12.27 | 0.016 |
| † Patient’s response in the “Characteristics of celiac disease questionnaire” to the question, “When you eat gluten what is the SINGLE most troubling symptom you develop or gets worse within 24 hours” were compared to their responses at 6 h after gluten challenge to the GloSS question, “What symptom/s overall have troubled you the most today, since having the food challenge? Please select a maximum of 3 symptoms. A. None; B. Abdominal Pain/cramps; C. Vomiting; D. Nausea; E. Diarrhea/loose stool; F. Gas (Flatulence); G. Bloating; H. Tiredness; I. Headache; J. Other (please specify)”. Patients were considered to have correctly predicted their worst symptom if any of the 3 responses to the GloSS question corresponded to the expected symptom after gluten. **‡**Overall, 283 stated a symptom, 3 did not know, 9 did not respond, and for 1 it was unclear if they correctly predicted their dominant symptom. †† Odds ratios and false discovery rate-adjusted 2-tail Fisher test p value for individual expected symptoms is for the 282 patients who stated any symptom they expected to be worst after gluten comparing patients expecting one symptom with others who expected any other another symptom. | | | | | |

| **Table S3:** Indication for testing, worst symptoms at diagnosis, and worst symptom expected after gluten exposure on gluten free diet, number (%) | | | | | | | | | | |
| --- | --- | --- | --- | --- | --- | --- | --- | --- | --- | --- |
| When you were first diagnosed what was the SINGLE most important reason you were tested for celiac disease? | |  | What was your SINGLE most troubling digestive symptom when you were diagnosed with celiac disease? | |  | What was your SINGLE most troubling non digestive symptom when you were diagnosed with celiac disease? | |  | When you eat gluten what is the SINGLE most troubling symptom you develop or gets worse within 24 hours? | |
| Iron deficiency | 45 (15) |  | Diarrhea | 83 (28) |  | Fatigue | 134(45) |  | Abdo. pain | 83 (28) |
| Abdo. pain | 44 (15) |  | Abdo. pain | 71 (24) |  | Headache | 39 (13) |  | Diarrhea | 66 (22) |
| Diarrhea | 38 (13) |  | Bloating | 53 (18) |  | Rash | 12 (4) |  | Vomiting | 39 (13) |
| Weight gain | 32 (11) |  | Vomiting | 15 (5) |  | Arthralgia | 5 (2) |  | Bloating | 31 (11) |
| Family History | 28 (9) |  | Nausea | 14 (5) |  | Iron deficiency | 5 (2) |  | Fatigue | 18 (6) |
| Fatigue | 27 (9) |  | Flatulence | 10 (3) |  | Depressed state | 4 (1) |  | Headache | 15 (5) |
| Bloating | 10 (3) |  | Constipation | 9 (3) |  | Anaemia | 3 (1) |  | Nausea | 13 (4) |
| Vomiting | 10 (3) |  | G-O reflux | 6 (2) |  | Migraine | 3 (1) |  | Flatulence | 6 (2) |
| Comorbidity | 9 (3) |  | GI sounds | 3 (1) |  | Other | 27 (9) |  | Other | 12 (4) |
| Rash | 7 (2) |  | Other | 5 (2) |  |  |  |  |  |  |
| Anemia | 4 (1) |  |  |  |  |  |  |  |  |  |
| Blood in stool | 4 (1) |  |  |  |  |  |  |  |  |  |
| Nausea | 4 (1) |  |  |  |  |  |  |  |  |  |
| G-O reflux | 4 (1) |  |  |  |  |  |  |  | Not known | 3 (1) |
| Other | 25 (8) |  | None | 24 (8) |  | None | 60 (20) |  | None | 3 (1) |
| Not stated | 4 (1) |  | Not stated | 2 (1) |  | Not stated | 3 (1) |  | Not stated | 5 (2) |

| **Table S4:** Correlations between peak severity of symptoms and IL-2 at 4 hours after gluten | | | | | | | | | | | |
| --- | --- | --- | --- | --- | --- | --- | --- | --- | --- | --- | --- |
|  | **FDR-adjusted 2-tail P-value** | | | | | | | | | | |
|  | GloSS | Nausea | Pain | Cramping | Bloating | Tiredness | Loose stool | Diarrhea | Gas | Headache | IL-2 4h |
| GloSS |  | <10^-15^ | <10^-15^ | <10^-15^ | <10^-15^ | <10^-15^ | <10^-15^ | <10^-15^ | 2.0x10^-11^ | 2.1x10^-6^ | 8.8x10^-12^ |
| Nausea | 0.82 |  | <10^-15^ | <10^-15^ | 1.1x10^-15^ | <10^-15^ | 1.5x10^-11^ | 5.4x10^-13^ | 9.2x10^-5^ | 4.8x10^-7^ | 4.3x10^-15^ |
| Pain | 0.72 | 0.55 |  | <10^-15^ | <10^-15^ | <10^-15^ | 1.1x10^-15^ | 1.2x10^-15^ | 2.3x10^-11^ | 7.4x10^-7^ | 8.6x10^-6^ |
| Cramping | 0.71 | 0.56 | 0.92 |  | <10^-15^ | <10^-15^ | 3.9x10^-15^ | 1.2x10^-15^ | 3.2x10^-13^ | 3.7x10^-8^ | 5.3x10^-6^ |
| Bloating | 0.61 | 0.45 | 0.63 | 0.60 |  | <10^-15^ | 5.9x10^-10^ | 2.3x10^-9^ | <10^-15^ | 2.1x10^-5^ | 0.03 |
| Tiredness | 0.56 | 0.54 | 0.52 | 0.54 | 0.48 |  | 2.1x10^-7^ | 4.7x10^-8^ | 5.6x10^-10^ | <10^-15^ | 0.18 |
| Loose stool | 0.49 | 0.39 | 0.45 | 0.44 | 0.35 | 0.30 |  | <10^-15^ | 2.8x10^-11^ | 0.0001 | 9.6x10^-5^ |
| Diarrhea | 0.48 | 0.41 | 0.45 | 0.45 | 0.34 | 0.32 | 0.80 |  | 2.6x10^-10^ | 3.1x10^-5^ | 3.7x10^-5^ |
| Gas | 0.38 | 0.23 | 0.38 | 0.41 | 0.53 | 0.36 | 0.38 | 0.36 |  | 8.6x10^-5^ | 0.43 |
| Headache | 0.28 | 0.29 | 0.29 | 0.32 | 0.25 | 0.51 | 0.22 | 0.24 | 0.23 |  | 0.93 |
| IL-2 4h | 0.39 | 0.44 | 0.26 | 0.27 | 0.13 | 0.08 | 0.23 | 0.24 | -0.05 | -0.01 |  |
|  | **Spearman correlation coefficient** | | | | | | | | | | |

| **Table S5:** Adverse events within 24 h after gluten challenge | | | | | |
| --- | --- | --- | --- | --- | --- |
| **Adverse event** ‡ | **Patients** | **Severity (n)** | | | **Onset** |
|  | n (%) | Mild | Moderate | Severe | Median (IQR) h:min |
| Any adverse event | 253 (86) | 73 | 143 | 37 |  |
| Nausea | 179 (61) | 68 | 84 | 27 | 1:24 (0:14-5:02) |
| Abdominal pain | 149 (51) | 64 | 77 | 8 | 1:50 (0:25-9:08) |
| Bloating | 134 (45) | 71 | 61 | 2 | 1:35 (0:14-4:57) |
| Headache | 114 (39) | 62 | 48 | 4 | 1:32 (0:18-6:41) |
| Fatigue | 97 (33) | 47 | 44 | 6 | 1:41 (0:13-7:20) |
| Diarrhoea | 96 (33) | 45 | 41 | 10 | 3:15 (0:52-21:07) |
| Vomiting | 62 (21) | 11 | 34 | 17 | 1:58 (0:58-3:41) |
| Flatulence | 52 (18) | 36 | 13 | 3 | 1:53 (0:14-9:34) |
| Feeling abnormal | 23 (8) | 10 | 13 | 0 | 1:39 (0:28-8:10) |
| Lethargy | 20 (7) | 3 | 14 | 3 | 2:06 (0:23-3:51) |
| Eructation | 17 (6) | 15 | 1 | 1 | 0:50 (0:05-4:58) |
| Dyspepsia | 12 (4) | 8 | 3 | 1 | 0:55 (0:18-4:48) |
| Dizziness | 10 (3) | 5 | 4 | 1 | 1:25 (0:27-5:00) |
| Gastrointestinal sounds abnormal | 9 (3) | 6 | 3 | 0 | 0:55 (0:31-3:53) |
| Constipation | 8 (3) | 4 | 4 | 0 | 4:53 (1:53-12:28) |
| Gastroesophageal reflux | 6 (2) | 3 | 3 | 0 | 1:55 (0:17-3:44) |
| Chills | 5 (2) | 2 | 3 | 0 | 1:54 (0:18-2:45) |
| Arthralgia | 5 (2) | 3 | 2 | 0 | 3:45 (1:35-7:50) |
| Facial flushing | 4 (1) | 3 | 0 | 1 | 0:39 (0:16-2:17) |
| Presyncope/syncope | 4 (1) | 2 | 2 | 0 | 2:40 (1:40-3:57) |
| Skin rash/itchiness | 4 (1) | 3 | 1 | 0 | 1:24 (0:46-18:28) |
| Decreased appetite | 3 (1) | 0 | 3 | 0 | 1:40 (0:46-3:27) |
| Aphthous ulcer | 2 (1) | 2 | 0 | 0 | 0:48 (0:48-0:48) |
| Vision blurred | 2 (1) | 1 | 1 | 0 | 2:09 (0:58-3:31) |
| ‡ MeDRA Preferred Terms (v21.0) were used to code adverse events, and their severity was graded according to Common Terminology Criteria for Adverse Events, Version 4.03. | | | | | |

| **Table S6**: Symptom severity and IL-2 after gluten according to patients’ characteristics | | | | | | | | | | |
| --- | --- | --- | --- | --- | --- | --- | --- | --- | --- | --- |
|  |  |  |  | **Peak GloSS** | |  | **Peak IL-2 serum concentration** | | | |
| Patient characteristics |  | Participants |  | Median (IQR) | |  | Median (IQR) pg/ml | |  | Responders (%) |
| Age (years) |  |  |  |  | |  |  | |  |  |
| 18 to 29 |  | 64 |  | 5 | (4 - 7) |  | 2.0 | (<0.5 - 7.5) |  | 41 (64) |
| 30 to 39 |  | 61 |  | 6 | (4 - 8) |  | 2.2 | (0.52 - 8.4) |  | 46 (75) |
| 40 to 49 |  | 69 |  | 6 | (3 - 8) |  | 4.3 | (1.3 - 15.3) |  | 59 (86) |
| 50 to 59 |  | 51 |  | 5 | (3 - 9) |  | 9.3 | (1.6 - 27.0) |  | 45 (88) |
| 60 to 70 |  | 50 |  | 5 | (3 - 6) |  | 4.0 | (0.7 - 21.4) |  | 41 (82) |
|  |  |  |  | Not significant ^a^ | |  | P = 0.00084 ^a^ | |  | P = 0.024 ^b^ |
| Gender | | | | | | | | | | |
| Female |  | 205 |  | 6 | (4 - 8) |  | 4.3 | (0.6 - 15.1) |  | 162 (79) |
| Male |  | 90 |  | 5 | (3 - 6) |  | 3.5 | (0.7 - 10.9) |  | 70 (78) |
|  |  |  |  | Not significant ^c^ | |  | Not significant ^c^ | |  | Not significant ^d^ |
| Height (cm) |  |  |  |  | |  |  | |  |  |
| 149 to 162 |  | 73 |  | 6 | (4 - 8) |  | 2.5 | (0.6 - 12.8) |  | 55 (75) |
| Over 162 to 168 |  | 74 |  | 6 | (4 - 7) |  | 4.0 | (0.7 - 15.3) |  | 58 (78) |
| Over 168 to 176 |  | 74 |  | 5 | (3 - 7.75) |  | 5.2 | (0.9 - 15.4) |  | 60 (81) |
| Over 176 to 194 |  | 74 |  | 5 | (3 - 6) |  | 3.3 | (1.1 - 9.3) |  | 59 (80) |
|  |  |  |  | Not significant ^a^ | |  | Not significant ^a^ | |  | Not significant ^b^ |
| Weight (kg) |  |  |  |  | |  |  | |  |  |
| 44.5 to 64.9 |  | 73 |  | 6 | (3 - 7) |  | 3.4 | (0.7 - 12.2) |  | 58 (79) |
| 64.9 to 75.1 |  | 74 |  | 5.5 | (4 - 8) |  | 4.9 | (1.1 - 15.4) |  | 63 (85) |
| 78.3 to 89.6 |  | 74 |  | 5 | (4 - 7) |  | 3.3 | (0.8 - 12.4) |  | 58 (78) |
| 89.7 to 152.7 |  | 73 |  | 6 | (4 - 7) |  | 2.2 | (<0.5 - 9.4) |  | 52 (71) |
|  |  |  |  | Not significant ^a^ | |  | Not significant ^a^ | |  | Not significant ^b^ |
| Age at diagnosis (years) |  |  |  |  | |  |  | |  |  |
| 0 to 9 |  | 11 |  | 6 | (3 - 6) |  | <0.5 | (<0.5 - 1.5) |  | 4 (36) |
| 10 to 19 |  | 34 |  | 5.5 | (4.25 - 7) |  | 1.5 | (<0.5 - 7.1) |  | 22 (65) |
| 20 to 29 |  | 55 |  | 6 | (4 - 8) |  | 5.3 | (1.3 - 10.5 |  | 46 (84) |
| 30 to 39 |  | 80 |  | 5 | (3 - 7) |  | 3.3 | (1.0 - 9.8) |  | 65 (81) |
| 40 to 49 |  | 60 |  | 6 | (3 - 8) |  | 7.8 | (1.1 - 31.2) |  | 51 (85) |
| 50 to 59 |  | 35 |  | 5 | (2.5 - 9) |  | 6.4 | (1.2 - 27.3) |  | 30 (86) |
| 60 to 69 |  | 18 |  | 4 | (2 - 5.75) |  | 2.6 | (0.5 - 11.7) |  | 13 (72) |
|  |  |  |  | Not significant ^a^ | |  |  | P = 0.018 ^a^ |  | Not significant ^b^ |
| Years from diagnosis |  |  |  |  | |  |  | |  |  |
| 1 to less than 2 |  | 23 |  | 5 | (3 - 6) |  | 2.1 | (<0.5 - 6.5) |  | 16 (70) |
| 2 to less than 3 |  | 42 |  | 6 | (3.25 - 8) |  | 1.7 | (<0.5 - 8.8) |  | 30 (71) |
| 3 to less than 4 |  | 22 |  | 5.5 | (3 - 6.75) |  | 3.9 | (0.6 - 10.4) |  | 17 (77) |
| 4 to less than 5 |  | 26 |  | 5.5 | (3.25 - 6) |  | 4.1 | (1.0 - 20.4) |  | 28 (88) |
| 5 to less than 7 |  | 41 |  | 5 | (3 - 7) |  | 5.6 | (1.1 - 24.5) |  | 36 (88) |
| 7 to less than 9 |  | 42 |  | 5.5 | (3 - 8) |  | 2.3 | (<0.5 - 12.7) |  | 29 (69) |
| 9 to less than 11 |  | 34 |  | 6 | (3 - 8) |  | 4.9 | (1.4 - 19.8) |  | 27 (79) |
| 11 to less than 16 |  | 35 |  | 5 | (4 - 8) |  | 4.6 | (1.6 - 17.0) |  | 32 (91) |
| 16 to 63 |  | 28 |  | 6 | (3 - 7) |  | 8.0 | (1.2 - 15.4) |  | 22 (79) |
|  |  |  |  | Not significant ^a^ | |  | Not significant ^a^ | |  | Not significant ^a^ |
| Serology |  |  |  |  | |  |  | |  |  |
| TG2 IgA & DGP IgG elevated |  | 5 |  | 4 | (1 - 4) |  | <0.5 | (<0.5 - <0.5) |  | 1 (20) |
| Only TG2 IgA elevated |  | 24 |  | 5.5 | (3 - 7) |  | 3.5 | (0.9 - 7.0) |  | 18 (75) |
| Only DGP IgG elevated |  | 23 |  | 4 | (3 - 6.5) |  | 2.5 | (1.0 - 7.7) |  | 18 (78) |
| TG2 IgA & DGP IgG normal |  | 240 |  | 5 | (3.75 - 8) |  | 4.6 | (0.7 - 23.0) |  | 192 (80) |
|  |  |  |  | Not significant ^e^ | |  | Not significant ^e^ | |  | Not significant ^b^ |
| Serum IgA levels |  |  |  |  | |  |  | |  |  |
| IgA deficiency (<7 mg/dl) |  | 2 |  | 4 | (4 - 4) |  | 1.3 | (1.2 - 1.5) |  | 2 (100) |
| IgA replete |  | 290 |  | 5 | (3 - 7.75) |  | 4.1 | (0.6 - 12.6) |  | 227 (78) |
| HLA-DQ2.5 |  |  |  |  | |  |  | |  |  |
| Present |  | 266 |  | 5.5 | (4 - 8) |  | 4.6 | (0.8 - 13.1) |  | 217 (82) |
| Absent |  | 29 |  | 5 | (3 - 6) |  | 0.6 | (<0.5 - 3.6) |  | 15 (52) |
|  |  |  |  | NS ^c^ | |  | P = 0.0061 ^c^ | |  | P = 0.0054 ^d^ |
| Country |  |  |  |  | |  |  | |  |  |
| United States |  | 126 |  | 6 | (3 - 7) |  | 4.8 | (0.9 - 15.3) |  | 100 (79) |
| New Zealand |  | 46 |  | 5 | (4 - 7) |  | 2.8 | (<0.5 - 11.4) |  | 31 (67) |
| Australia |  | 123 |  | 5 | (3 - 8) |  | 3.6 | (0.7 - 11.1) |  | 101 (82) |
|  |  |  |  | NS ^e^ | |  | NS ^e^ | |  | NS ^f^ |
| P-value calculated according to: ^a^ Spearman's rank correlation coefficient, ^b^ Cochran–Armitage test for trend, ^c^ Mann–Whitney U test, ^d^ Fisher's exact test, ^e^ Kruskal-Wallis H test, or ^f^ chi-square test. | | | | | | | | | | |

| **Table S7:** Peak symptom severity after gluten according to HLA-DQ genotype | | | | | | | | | | | |
| --- | --- | --- | --- | --- | --- | --- | --- | --- | --- | --- | --- |
| Genotype | Patients | Peak symptom severity during the six hours after gluten challenge, median (interquartile range) rated 0-10 | | | | | | | | | |
|  | N | Global | Cramping | Abdo. pain | Bloating | Diarrhoea | Gas | Loose stool | Nausea | Headache | Tiredness |
| DQ2.5 positive | 266 | 6 (3-8) | 4 (1-6) | 4 (2-6) | 4 (2-6) | 0 (0-3) | 2 (1-4) | 0 (0-3) | 5 (2-8) | 3 (1-5) | 5 (3-7) |
| DQ2.5 negative‡ | 29 | 5 (3-6) | 2 (1-5) | 3 (1-5) | 4 (2-6) | 0 (0-4) | 3 (2-5) | 1 (0-4) | 3 (1-5) | 3 (1-5) | 5 (3-7) |
| **HLA-DQ2.5 subgroups†** | | | | | | | | | | | |
| DQ2.5, 2.5 | 34 | 5 (3-7) | 3 (1-5) | 3 (1-5) | 4 (2-6) | 0 (0-1) | 2 (1-4) | 0 (0-3) | 4 (1-7) | 3 (1-5) | 5 (2-7) |
| DQ2.5, 2.2 | 56 | 6 (3-8) | 4 (1-6) | 4 (2-6) | 4 (2-6) | 0 (0-3) | 2 (1-4) | 0 (0-3) | 5 (2-8) | 3 (1-5) | 5 (3-7) |
| DQ2.5, 7 | 15 | 6 (4-7) | 3 (1-6) | 4 (2-6) | 4 (2-7) | 0 (0-3) | 2 (2-5) | 1 (0-5) | 4 (3-8) | 3 (1-6) | 5 (2-8) |
| DQ2.5, 8 | 36 | 6 (3-7) | 3 (2-5) | 3 (2-6) | 3 (2-5) | 0 (0-1) | 2 (1-3) | 0 (0-2) | 4 (3-7) | 2 (0-4) | 6 (4-7) |
| DQ2.5trans | 18 | 5 (4-8) | 3 (1-4) | 3 (1-6) | 4 (1-7) | 0 (0-0) | 1 (1-4) | 0 (0-2) | 5 (2-7) | 3 (0-4) | 4 (2-7) |
| DQ2.5cis, X | 107 | 6 (4-8) | 4 (1-6) | 4 (1-6) | 4 (2-6) | 0 (0-3) | 2 (1-5) | 0 (0-3) | 5 (2-8) | 4 (1-6) | 6 (3-7) |
| ‡ Genotypes, symptoms and IL-2 responses for patients negative for both *HLA-DQA1*05* & *HLA-DQB1*02* are listed in Table S5.  † *HLA-DQA* & *DQB* alleles for DQ2.5, 2.5: *05* & *02*; DQ2.5, 2.2: *02,05* & *02*; DQ2.5, 7: *05* & *02,03*; DQ2.5, 8: *03,05* & *02,03*; DQ2.5cis: 05,other (not *02* or *03*)  & *02*,other (not *03*); DQ2.5trans/DQ2.2, 7: *02,05* & *02,03* | | | | | | | | | | | |

| **Table S8:** Symptoms and serum IL-2 after gluten in 29 participants negative for HLA-DQ2.5 (*DQA1*05* and *DQB1*02*) | | | | | | | | | | | | | | | | | |
| --- | --- | --- | --- | --- | --- | --- | --- | --- | --- | --- | --- | --- | --- | --- | --- | --- | --- |
| HLA-DQ alleles | | Peak hourly description & rating for global digestive symptoms (GloSS) and peak hourly rating for individual symptoms (modified CeD PRO) (0 – 10) | | | | | | | | | | | IL-2 serum concentration (pg/ml) | | | | |
| *DQA* | *DQB* | Global | | Cr | AP | Bl | Di | Gas | LS | Na | He | Ti | BSL | 2 h | 4 h | 6 h | Max. |
| HLA-DQ8 homozygous | | | | | | | | | | | | | | | | | |
| *03:01, 03* | *03, 03* | Severe | 7 | 6 | 6 | 8 | 8 | 6 | 10 | 4 | 6 | 8 | < 0.5 | 2.6 | 0.7 | < 0.5 | 2.6 |
| *03:01, 03* | *03, 03* | Mild | 5 | 2 | 1 | 3 | 0 | 5 | 0 | 3 | 5 | 10 | < 0.5 | < 0.5 | 0.6 | < 0.5 | 0.6 |
| *03:01, 03* | *03, 03* | Very mild | 1 | 0 | 0 | 1 | 0 | 1 | 1 | 0 | 1 | 0 | < 0.5 | < 0.5 | < 0.5 | < 0.5 | < 0.5 |
| HLA-DQ8 and DQ2.2 | | | | | | | | | | | | | | | | | |
| *02:01, 03* | *02, 03* | Moderate | 5 | 4 | 3 | 6 | 6 | 6 | 7 | 2 | 1 | 2 | 6.1 | 14.7 | 24.2 | 13.8 | 24.2 |
| *02:01, 03* | *02, 03* | Mild | 5 | 0 | 1 | 5 | 0 | 0 | 0 | 1 | 0 | 0 | < 0.5 | < 0.5 | 3.0 | 5.9 | 5.9 |
| *02:01, 03* | *02, 03* | Mild | 3 | 2 | 0 | 4 | 0 | 1 | 0 | 3 | 0 | 4 | < 0.5 | 2.0 | 3.9 | 3.8 | 3.9 |
| *02:01, 03* | *02, 03* | Mild | 3 | 2 | 1 | 2 | 0 | 3 | 0 | 6 | 2 | 6 | < 0.5 | 1.2 | 3.6 | 1.8 | 3.6 |
| *02:01, 03* | *02, 03* | V severe | 8 | 8 | 8 | 9 | 8 | 7 | 8 | 8 | 5 | 8 | < 0.5 | 0.8 | 1.5 | 0.7 | 1.5 |
| *02:01, 03* | *02, 03* | Mild | 5 | 5 | 5 | 7 | 7 | 4 | 0 | 10 | 4 | 10 | < 0.5 | < 0.5 | < 0.5 | < 0.5 | < 0.5 |
| HLA-DQ8 and DQ2.3 | | | | | | | | | | | | | | | | | |
| *03:01, 03* | *02, 03* | Moderate | 5 | 3 | 4 | 5 | 0 | 3 | 0 | 1 | 1 | 4 | < 0.5 | < 0.5 | 0.7 | < 0.5 | 0.7 |
| HLA-DQ8 and DQ7 | | | | | | | | | | | | | | | | | |
| *03:01, 05* | *03, 03* | Moderate | 5 | 5 | 5 | 4 | 4 | 4 | 6 | 3 | 7 | 7 | < 0.5 | 1.0 | 2.9 | 1.1 | 2.9 |
| *03:01, 05* | *03, 03* | Mild | 3 | 0 | 1 | 0 | 1 | 1 | 1 | 3 | 4 | 4 | < 0.5 | < 0.5 | < 0.5 | < 0.5 | < 0.5 |
| HLA-DQ8 and other | | | | | | | | | | | | | | | | | |
| *01, 03* | *03, 06* | Moderate | 6 | 2 | 2 | 3 | 3 | 4 | 4 | 7 | 0 | 3 | < 0.5 | 26.3 | 31.7 | 14.1 | 31.7 |
| *03, 04:01* | *03, 04* | Moderate | 6 | 4 | 3 | 3 | 6 | 6 | 5 | 2 | 5 | 5 | < 0.5 | 4.6 | 1.6 | < 0.5 | 4.6 |
| *01, 03* | *03, 05* | Mild | 5 | 1 | 2 | 2 | 4 | 3 | 4 | 2 | 0 | 0 | < 0.5 | < 0.5 | < 0.5 | < 0.5 | < 0.5 |
| *01, 03* | *03, 05* | Mild | 3 | 2 | 2 | 3 | 0 | 0 | 1 | 3 | 4 | 4 | < 0.5 | < 0.5 | < 0.5 | < 0.5 | < 0.5 |
| *01, 03* | *03, 06* | Moderate | 6 | 5 | 5 | 5 | 1 | 4 | 1 | 5 | 5 | 6 | < 0.5 | < 0.5 | < 0.5 | < 0.5 | < 0.5 |
| *01, 03* | *03, 06* | Very mild | 3 | 1 | 1 | 2 | 2 | 2 | 0 | 0 | 8 | 9 | < 0.5 | < 0.5 | < 0.5 | < 0.5 | < 0.5 |
| *01, 03* | *03, 06* | Moderate | 6 | 5 | 7 | 7 | 0 | 5 | 3 | 1 | 0 | 2 | < 0.5 | < 0.5 | < 0.5 | < 0.5 | < 0.5 |
| *01, 03* | *03, 06* | Moderate | 3 | 1 | 4 | 2 | 2 | 2 | 3 | 1 | 4 | 5 | < 0.5 | < 0.5 | < 0.5 | < 0.5 | < 0.5 |
| *01, 03* | *03, 06* | Severe | 7 | 7 | 5 | 8 | 0 | 8 | 2 | 7 | 2 | 8 | < 0.5 | < 0.5 | < 0.5 | < 0.5 | < 0.5 |
| *01, 03* | *03, 06* | Moderate | 5 | 2 | 2 | 5 | 0 | 0 | 0 | 5 | 6 | 6 | < 0.5 | < 0.5 | < 0.5 | < 0.5 | < 0.5 |
| HLA-DQ2.2 homozygous | | | | | | | | | | | | | | | | | |
| *02:01, 02:01* | *02, 02* | Mild | 2 | 0 | 1 | 2 | 0 | 1 | 0 | 0 | 3 | 3 | < 0.5 | 9.5 | 42.8 | 11.8 | 42.8 |
| HLA-DQ2.2 and other | | | | | | | | | | | | | | | | | |
| *02:01, 03* | *02, 04* | Severe | 8 | 7 | 6 | 3 | 8 | 6 | 8 | 1 | 1 | 2 | < 0.5 | < 0.5 | 1.9 | 0.8 | 1.9 |
| *01, 02:01* | *02, 06* | Moderate | 6 | 5 | 5 | 5 | 0 | 2 | 0 | 4 | 5 | 6 | < 0.5 | < 0.5 | 1.1 | < 0.5 | 1.1 |
| *01, 02:01* | *02, 06* | Mild | 3 | 0 | 0 | 3 | 0 | 3 | 0 | 2 | 0 | 3 | < 0.5 | < 0.5 | < 0.5 | < 0.5 | < 0.5 |
| HLA-DQ2.3 and other | | | | | | | | | | | | | | | | | |
| *01, 03* | *02, 05* | Very mild | 1 | 1 | 0 | 0 | 0 | 4 | 0 | 0 | 3 | 8 | < 0.5 | < 0.5 | < 0.5 | < 0.5 | < 0.5 |
| HLA-DQ7 and other | | | | | | | | | | | | | | | | | |
| *01, 05* | *03, 06* | V. severe | 10 | 7 | 7 | 7 | 2 | 5 | 2 | 9 | 1 | 6 | < 0.5 | 234.9 | 27.8 | 2.8 | 234.9 |
| *01, 05* | *03, 06* | Moderate | 6 | 7 | 6 | 8 | 0 | 7 | 0 | 4 | 1 | 6 | < 0.5 | < 0.5 | < 0.5 | < 0.5 | < 0.5 |
| Abbreviations: cramping, Cr; abdominal pain, AP; bloating, Bl; diarrhea, Di; gas/flatulence, Gas; loose stools, LS; nausea, Na; headache, He; tiredness, Ti; baseline, BSL; | | | | | | | | | | | | | | | | | |

| **Table S9:** Study Independent Ethics Committees and approvals |
| --- |
| *United States of America* |
| 1. Copernicus Group IRB, 5000 Centre Green Way, Suite 200, Cary NC, 27513, Study Number 20181291 Approval date: 17 Jul 2018 2. Columbia University IRB, 154 Haven Avenue, 1st Floor, New York NY, 10032, Study Number IRB-AAAR9068 Approval date: 04 Jan 2019 3. Mayo Clinic IRB, 200 First Street SW, Rochester MN, 55905, Study Number 18-004575 Approval date: 16 Jan 2019 4. The University of Chicago IRB, 5841 South Maryland Avenue, MC7132, I-625, Chicago IL, 60637, Study Number IRB18-1200 Approval date: 31 Jan 2019 |
| *New Zealand* |
| 1. Central Health and Disability Ethics Committee, Ministry of Health, 133 Molesworth St, Thorndon, Wellington, 6011, Study Number 18/SCOTT/70 Approval date: 06 Sep 2018 |
| *Australia* |
| 1. Bellberry Limited, 129 Glen Osmond Road, Eastwood SA, 5063, Application Number 2018-07-562-A-13 Approval date: 20 Sep 2018 2. Melbourne Health Human Research Ethics Committee, Office for Research, Level 2 South West, 300 Grattan Street, Parkville VIC, 3050, Study Number HREC/43048/MH-2018 Approval date: 08 Oct 2018 3. Uniting Care Health, 129 Glen Osmond Road, Eastwood SA, 5063, Study Number 1818 Approval date: 22 Oct 2018 |
|  |
| **Figure S1:** Patient disposition. |
